# Reducing Recurrent Urinary Tract Infections in Women with MV140 Impacts Personal Burden of Disease: Secondary Analyses of a Randomized Placebo-controlled Efficacy Study

**DOI:** 10.1016/j.euros.2024.03.010

**Published:** 2024-03-29

**Authors:** J. Curtis Nickel, Stephen Foley, Bob Yang, Miguel Casanovas, Raquel Caballero, Carmen María Diez-Rivero, María-Fernanda Lorenzo-Gómez

**Affiliations:** a Queen’s University, Kingston, ON, Canada; b Royal Berkshire Hospital, Reading, UK; c Inmunotek S.L., Alcalá de Henares, Madrid, Spain; d University Hospital of Salamanca, Salamanca, Spain

**Keywords:** Urinary tract infection, Quality of life, Burden, Cystitis, Vaccine, Women, Antibiotics, Prophylaxis

## Abstract

**Background:**

Recurrent urinary tract infection (rUTI) remains a major health burden for women. A randomized, double-blind, placebo-controlled trial (RCT; NCT02543827) reported that female patients with rUTI receiving a sublingual vaccine, MV140, had a reduction in rUTI and increase in UTI-free rate compared with placebo.

**Objective:**

To determine the impact of MV140 on the personal burden of disease in women with rUTI using secondary endpoint data from the pivotal RCT evaluating MV140.

**Design, setting, and participants:**

In the primary RCT, female patients with rUTI enrolled in Spain and UK (from October 2015 to April 2019) were randomized to placebo (6 mo) or MV140 (3 or 6 mo), and followed for 12 mo. Individuals analyzed in this secondary analysis included those in the placebo and 3-mo (recommended dose) groups.

**Intervention:**

A polybacterial sublingual vaccine, MV140 (four inactivated whole-cell bacteria—*Escherichia coli*, *Klebsiella pneumoniae*, *Proteus vulgaris*, and *Enterococcus faecalis*), or placebo.

**Outcome measurements and statistical analysis:**

Symptom severity scoring, antibiotic use, safety, and multiple aspects of quality of life (QoL; Short-Form Questionnaire [SF-36]) were assessed.

**Results and limitations:**

Compared with the placebo group (*n* = 76), the 3-mo vaccinated group (*n* = 74) experienced fewer overall UTI symptoms (mean symptom score 102.2 ± 222.9 vs 194.2 ± 178.8; *p* = 0.0002), fewer days on antibiotics (12.4 ± 17.7 vs 28.7 ± 25.2; *p* = 0.0001), and improved total, general, and physical SF-36 QoL improvement (differences in means for total SF-36 score 15.7; 95% confidence interval [CI] 8.80, 22.64; *p* < 0.0001), with only social function QoL showing no impact (4.07; 95% CI –4.93, 13.08; *p* = 0.3744).

**Conclusions:**

Three months of MV140 is associated with a reduction of the personal burden of UTI by reducing overall UTI symptoms and antibiotic use, improving QoL in women with rUTI.

**Patient summary:**

Three months of MV140 vaccine, which has previously been shown to reduce the risk of urinary tract infection safely, is associated with a reduction in the personal burden of disease.

## Take Home Message

A secondary analysis of a randomized placebo-controlled trial of MV140 vaccine demonstrating a reduction of recurrent urinary tract infection (UTI) in women showed an associated reduction in the personal burden of disease with a reduction of UTI symptoms and antibiotics, and improvement in quality of life.

## 1 Introduction

Urinary tract infections (UTIs) are one of the most common bacterial infections worldwide, particularly in women [1], [2]. Recurrent UTIs (rUTIs), defined as experiencing three or more UTIs over a 12-mo period (or two or more UTIs over a 6-mo period) [3], [4] can be a severely debilitating condition. The personal impact on the individual suffering from rUTIs includes the acute pain and urinary symptoms associated with each episode, potential development of a more serious UTI (eg, pyelonephritis), disability (time off work and life activities), deterioration in psychosocial parameters (eg, depression and anxiety), and the very real and serious side effects of taking antibiotics (short term for treatment and long term for prophylaxis) [5], [6], [7], [8], [9], [10], [11]. These effects on their social and daily lives result in a physical and social burden of a chronic disease resulting in deterioration of quality of life (QoL) [9], [10], [11]. Women with rUTIs reported, in focus [12] and web-based [6] discussion groups, of the emotional and daily life disruptions, and that they are fearful of the adverse effects of antibiotics and are frustrated with the medical profession for not addressing these fears and optimizing antibiotic stewardship [12].

Antibiotic management results in some improvement in QoL [13], [14], but reinfection rates are high [3], and persistence of disease in patients with rUTIs, particularly those with antibiotic resistance [13] suggests that effective antibiotic-based treatment strategies remain problematic in clinical practice [8]. Management of rUTIs in women is one of the most significant factors leading to antibiotic prescriptions, and this massive use of antibiotics has contributed to increasing overall antibiotic resistance [15], [16].

The European [4] but not the American Urological Association [3] guidelines recommend an immunoactive (vaccine) strategy for preventing rUTIs. A nonrandomized observational study with oral bacterial lysate therapy OM-89 [9] showed a measurable decrease in anxiety and depression when used as a prophylaxis for rUTIs. A recently completed randomized placebo-controlled trial showed that a novel sublingual vaccine, MV140 [17], confirmed previous observational reports [18] that MV140 reduced the risk of UTIs significantly in women suffering from rUTIs. While overall QoL was shown to improve along with an associated lower risk of UTIs, a secondary analysis of the personal burden and comprehensive breakdown of QoL data collected as part of the study design can allow determination of the impact of a nonantibiotic intervention to prevent or reduce UTIs using the clinically recommended 3-mo dosing schedule on specific QoL domains and other disease burden parameters in women with rUTIs.

## 2 Patients and methods

The primary efficacy and safety study, an international phase 3 multicenter, randomized, double-blind, placebo-controlled, parallel-group clinical trial (RCT; EUDRACT 2013-001838-17, NCT02543827) enrolled women (18–75 yr old) diagnosed with rUTIs (at least five uncomplicated cystitis in the previous year). The exclusion criteria included complicated UTIs, comorbidities associated with the genitourinary tract, and/or immunological diseases.

Following randomization (allocated by means of a list generated by Random software in a 1:1:1 ratio), participants received placebo for 6 mo, MV140 for 3 mo (plus 3 mo of placebo), or MV140 for 6 mo. Trial personnel and participants were masked to treatment assignments during recruitment and follow-up. The active product (MV140; Inmunotek S.L., Madrid, Spain) consists of a suspension of whole-cell heat-inactivated bacteria (300 Formazin Turbidity Units) in glycerol, sodium chloride, artificial pineapple flavoring, and water. Included are equal percentages of selected strains of four bacterial species (*Escherichia coli* V121, *Klebsiella pneumoniae* V113, *Enterococcus faecalis* V125, and *Proteus vulgaris* V127). The placebo preparation contained all ingredients as MV140 except for the bacteria. The assigned treatment was administered daily sublingually by applying two sprays of 100 µl each under the tongue. All participants were followed for a full study period of 12 mo from randomization. Clinic visits were arranged every 3 mo. Comprehensive details including rules for defining UTIs, prescribing antibiotics, safety assessment, and trial oversight are described in the primary RCT publication [17], and the full trial protocol is available at evidence.nejm.org.

The trial was conducted in accordance with Good Clinical Practice guidelines and the provisions of the Declaration of Helsinki. The trial was designed and sponsored by Inmunotek S.L., which provided the study drug. The protocol and amendments were approved by the institutional review boards or independent ethics committees at each study site and national regulatory authorities. Written informed consent was obtained from all participants.

The primary and major secondary outcomes of the published study were, respectively, the number of UTI episodes and proportion of participants remaining UTI free in the 9-mo efficacy period. The secondary patient burden outcomes for this present study included UTI symptom analysis (total days with symptoms and total symptom score during UTIs), days with antibiotics, and QoL assessment (total and subscores of the Short-Form Questionnaire [SF-36] questionnaire) [19], [20]. To our knowledge, the minimal clinically important difference in SF-36 scores in female patients with rUTIs has yet to be determined. Symptom scoring was documented daily by patient’s diary on a scale of 0–3: 0 = no obvious symptoms; 1 = a little (mild, trivial, clearly present, but not bothersome); 2 = quite a lot (moderate, bothersome, but not disabling or intolerable); and 3 = very much (severe, disabling, and/or intolerable). The analysis was performed using the intention-to-treat (ITT) population (placebo, 76 patients and MV140 3-mo, 74 patients). For the analysis, results were rated per day, per UTI episode, and for the whole study period. Following database lock, missing data were corrected with the last observation carried forward. The “symptom score during UTI” was calculated as the sum of the overall symptom data for each UTI, while the “daily symptom score during UTI” was calculated by summing the daily score value for the days the patient had symptoms. No minimal clinically important difference for this questionnaire is available.

The primary burden analyses in this secondary substudy comparing the placebo group with the group receiving the clinically recommended 3-mo dose were carried out by the protocol-defined ITT population (all participants assigned randomly to treatment who completed week 12 according to the treatment assignment at randomization). For these ITT analyses, missing data were imputed with the last observation carried forward. Further detail on sample size calculation, evaluable populations, and overall statistical analysis can be found in the study of Lorenzo-Gómez et al [17] and evidence.nejm.org. Differences in days with symptoms and total symptom scores as well as days on antibiotics were calculated by the Mann-Whitney-Wilcoxon test with Bonferroni correction. Differences between groups in daily symptom scores during UTIs were calculated by the Kruskal-Wallis nonparametric test. The data provided in the SF-36 QoL questionnaire completed by each participant were transferred to an online calculator (https://orthotoolkit.com/sf-36/), which provides a scale of 1–100 as well as the mean (and median) for each of the nine domains of the questionnaire. Comparisons between SF-36 total scores and subscores were analyzed by a pre/post comparison of differences between means by a mixed-effect model. The level of significance of *p* < 0.05 was established for all tests performed (two sided), following correction for multiple comparisons.

## 3 Results

**Fig. 1:**
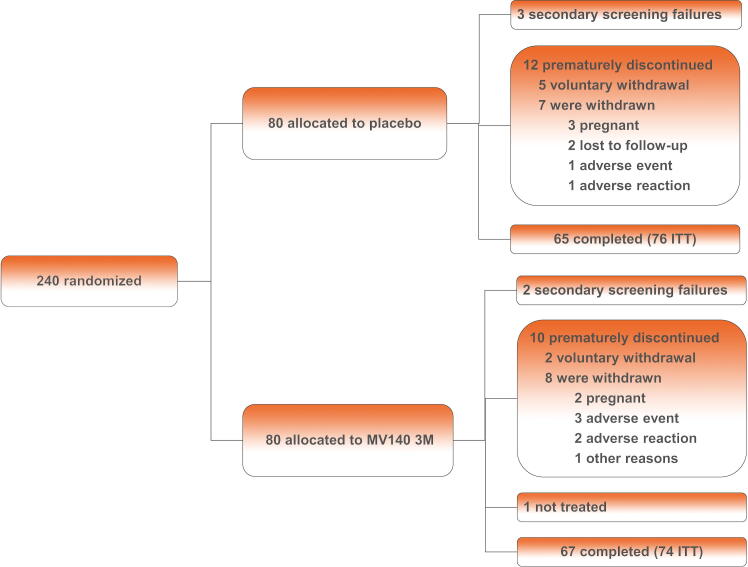
Randomization and patient flow diagram for the two relevant arms of the RCT: the placebo and 3-mo treatment groups. The third arm, the 6-mo treatment arm, was not included in this patient burden analysis. The official study CONSORT diagram is available in the primary publication [17]. ITT = intention to treat; RCT = randomized controlled trial.

Of the 392 women screened, a total of 230 (78 placebo group, 77 MV140 3-mo group, and 75 MV140 6-mo group) initiated sublingual vaccination and were included in the primary publication safety analysis report [17]. A total of 150 (76 placebo and 74 MV140 3 mo) patients were included in the ITT population for this subgroup analysis (CONSORT diagram is available for the entire study; Figure 1 describes the relevant flow diagram for this subanalysis). A detailed description of the demographic and clinical characteristics is available in the primary report [17]. The mean (standard deviation) age of the placebo and MV140 3-mo groups was 47.9 (17.2) and 51.1 (16.8), respectively (*p* = 0.246, Kruskal-Wallis nonparametric test), with 35 (46.1%) and 38 (45.7%), respectively, reporting premenopausal status (*p* = 0.4421, chi-square test comparing total premenopausal vs postmenopausal). The prestudy median number of UTIs was 6.0 (interquartile range [IQR] 5.0–6.0 and 5.0–7.0 for the placebo and 3-mo groups, respectively). The median UTI episodes and UTI-free rate in each group during the efficacy period have been reported in the primary RCT publication [17] and described in the Discussion section.

**Fig. 2:**
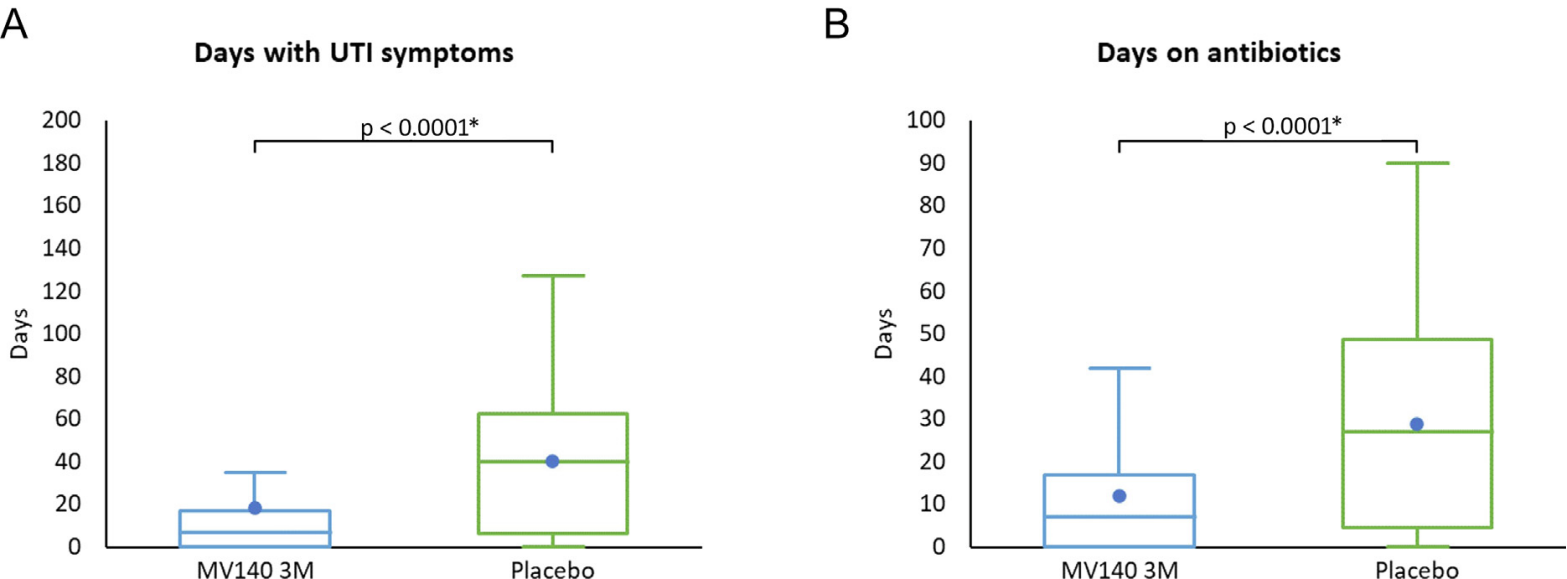
Comparison of UTI symptoms and antibiotic use between the recommended 3-mo dose of MV140 and placebo during the 9-mo efficacy period. (A) Days with UTI symptoms are shown as box plots, indicating the mean (dot), median, interquartile range, minimum, and maximum. (B) Days on antibiotics are shown as box plots, indicating the mean (dot), median, interquartile range, minimum, and maximum. * *p* < 0.0001, Mann-Whitney-Wilcoxon test comparing the placebo group with the 3-mo MV140 (MV140 3M) group. UTI = urinary tract infection.

Compared with the placebo group, participants in the 3-mo MV140 group experienced significantly fewer days with symptoms (19.2 ± 36.4 vs 40.1 ± 35.5; *p* < 0.0001), total overall symptoms from UTIs (symptom scoring 102.2 ± 222.9 vs 194.2 ± 178.8; *p* = 0.0002), but not symptoms suffered during a UTI episode (daily mean symptom score during UTIs 3.1 ± 2.9 vs 3.8 ± 3.1 ± 2.7; *p* = 0.387). MV140 therapy resulted in significantly fewer days on antibiotics (12.4 ± 17.7 vs 28.7 ± 25.2; *p* = 0.0001; Fig. 2 and Table 1).

**Table 1:** Differences between means in UTI symptoms and antibiotic use outcomes from baseline to 12 mo in the ITT population

Patient burden outcome	Placebo		Months 0–12
		MV140 3M	*p* value vs placebo
Total days with symptoms, mean (SD)	40.1 (35.5)	19.2 (36.4)	<0.0001 a
Total symptom score during UTIs, mean (SD)	194.3 (178.8)	102.2 (222.9)	0.0002 a
Daily symptom score during UTIs, mean (SD)	3.8 (2.7)	3.1 (2.9)	0.387 b
Days with antibiotics, mean (SD)	28.7 (25.2)	12.4 (17.7)	0.0001 a

ITT = intention to treat; SD = standard deviation; UTI = urinary tract infection.

a Mann-Whitney-Wilcoxon (test with Bonferroni correction).

b Kruskal-Wallis nonparametric test.

**Fig. 3:**
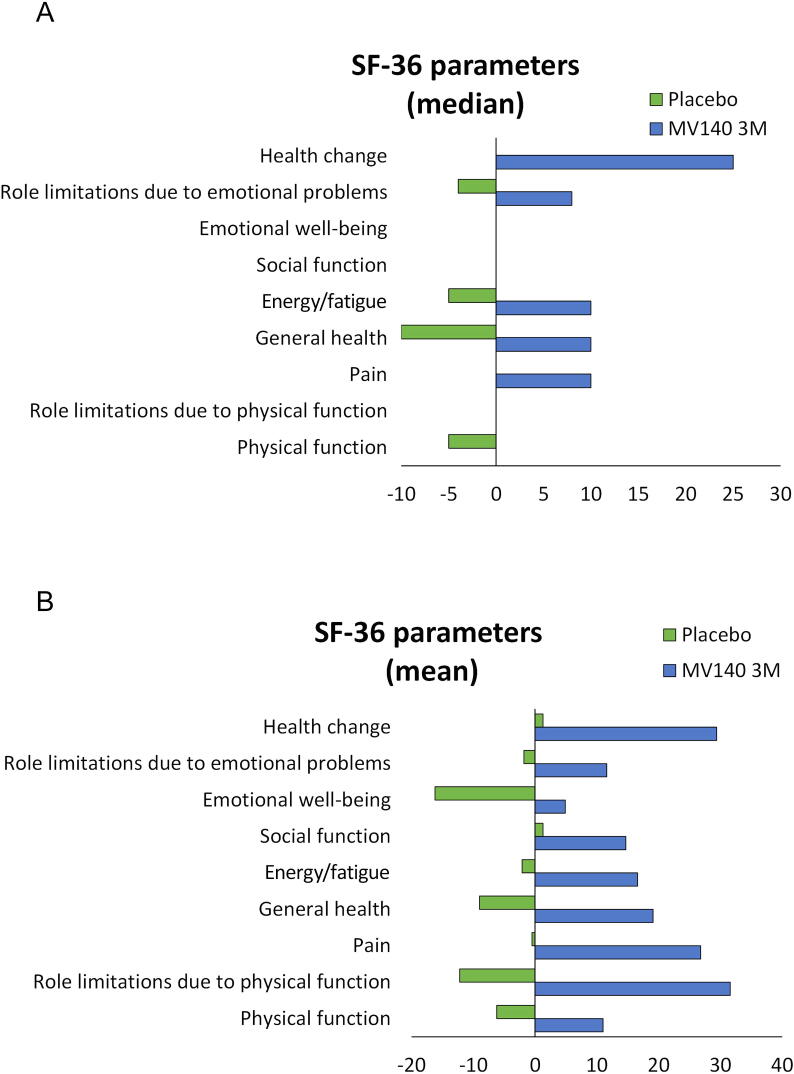
Changes in quality of life throughout the whole study period for participants on the recommended 3-mo dose of MV140 and placebo. Scores are from the 36-item Short-Form Questionnaire (SF-36); higher scores indicate better health status. The (A) median and (B) mean changes from baseline are shown for each of the parameters included in the questionnaire.

The median SF-36 QoL scores were balanced at baseline between the placebo (65.7 [IQR, 52.2–79.2]) and MV140 3-mo (67.5 [IQR, 45.3–77.0]) groups. Compared with the placebo group (which showed no change in QoL from baseline), the MV140 3-mo group noted a significant improvement in the total SF-36 score and almost all the SF-36 subscores over time (Fig. 3 and Table 2). Table 3 describes the differences between means in secondary personal burden outcomes (including SF-36 subscores) from baseline to 3, 6, 9, and 12 mo in the ITT population. The difference between means for the 0–12 mo time period was significant (15.7; 95% confidence interval [CI] 8.80, 22.64; *p* < 0.0001). Furthermore, there was a significant difference between the 3-mo MV140 and placebo groups in the change of the physical function, role limitations due to physical problems, pain, general health, energy/fatigue, and role limitations due to emotional problems (all significant differences between means reported in Table 3), but not in social function in the SF-36 subscores for the 12 mo of the study (differences between means 4.07; 95% CI –4.93, 13.08; *p* = 0.3744). Figure 3 shows graphically the differences (mean and median) between the group that received the clinically recommended dose (3 mo) and the placebo group.

**Table 2:** Secondary personal burden SF-36–related outcomes (mean and standard deviation [SD]) at baseline and 3, 6, 9, and 12 mo in the ITT population

Patient burden outcome	Baseline		3 mo		6 mo		9 mo		12 mo	
	Placebo	MV140 3M	Placebo	MV140 3M	Placebo	MV140 3M	Placebo	MV140 3M	Placebo	MV140 3M
SF-36 total score, mean (SD)	64.2 (17.3)	61.1 (20.9)	61.2 (19.3)	66.2 (15.5)	62.2 (20.2)	71.1 (19.4)	56.9 (19.9)	71.9 (20.4)	61.1 (21.7)	75.6 (18.1)
SF-36 physical function, mean (SD)	80.6 (22.0)	76.2 (27.2)	78.2 (24.9)	77.2 (24.0)	77.8 (23.3)	81.9 (23.9)	74.5 (24.4)	82.6 (24.7)	76.8 (24.0)	82.3 (21.7)
SF-36 role limitations due to physical problems, mean (SD)	63.3 (40.8)	60.1 (45.0)	51.5 (44.0)	64.8 (41.2)	55.6 (45.8)	76.7 (39.7)	48.2 (44.0)	76.2 (39.4)	50.6 (44.7)	76.7 (38.3)
SF-36 pain, mean (SD)	60.0 (25.4)	57.6 (26.7)	60.4 (23.9)	62.2 (22.1)	59.2 (26.1)	66.9 (25.1)	52.0 (21.8)	69.2 (25.2)	60.8 (24.3)	73.9 (22.3)
SF-36 general health, mean (SD)	54.4 (19.4)	51.0 (19.0)	52.0 (21.6)	55.3 (16.9)	49.8 (21.2)	57.6 (18.0)	46.0 (20.8)	59.2 (17.5)	46.2 (23.7)	59.9 (22.8)
SF-36 energy/fatigue, mean (SD)	53.8 (17.8)	50.9 (21.9)	51.5 (19.7)	55.3 (16.6)	51.0 (20.6)	57.9 (19.8)	48.6 (20.4)	58.5 (19.3)	53.1 (19.9)	62.1 (22.4)
SF-36 social function, mean (SD)	77.1 (21.7)	74.7 (24.1)	75.5 (20.9)	78.3 (18.3)	76.2 (22.7)	84.2 (19.3)	71.2 (24.5)	84.3 (18.8)	78.8 (20.2)	86.1 (19.5)
SF-36 emotional well-being, mean (SD)	73.9 (40.8)	65.5 (44.5)	64.7 (42.4)	73.5 (37.8)	76.3 (37.4)	77.8 (40.2)	65.1 (43.5)	75.4 (41.0)	66.7 (43.6)	89.9 (27.7)
SF-36 role limitations due to emotional problems, mean (SD)	69.1 (16.6)	62.8 (19.7)	66.2 (18.4)	68.3 (15.6)	64.7 (20.5)	69.1 (18.7)	61.7 (20.2)	72.9 (16.1)	68.4 (17.8)	76.4 (17.3)
SF-36 health change, mean (SD)	45.0 (18.3)	49.1 (20.6)	52.0 (22.3)	54.0 (17.8)	49.4 (21.0)	67.2 (21.2)	44.6 (17.1)	69.6 (23.8)	48.2 (24.9)	70.3 (29.0)

ITT = intention to treat; SF-36 = Short-Form Questionnaire.

**Table 3:** Differences between means in secondary personal SF-36 burden outcomes from baseline to 3, 6, 9, and 12 mo in ITT population (unless otherwise specified)

Patient burden outcome	Placebo	Months 0–12		Months 0–3		Months 0–6		3M months 0–9	
		MV140 3M	*p*-value vs placebo a	MV140 3M	*p* value vs placebo a	MV140 3M	*p* value vs placebo a	MV140	*p* value vs placebo a
SF-36 total score differences between means (95% CI)	–	15.72 (8.80, 22.64)	<0.0001	4.44 (–2.19, 11.07)	0.1889	10.62 (3.80, 17.45)	0.0024	15.43 (8.48, 22.38)	<0.0001
SF-36 physical function differences between means (95% CI)	–	8.21 (1.33, 15.09)	0.0195	5.99 (–0.84, 12.82)	0.0854	5.99 (–0.84, 12.82)	0.0854	8.16 (1.27, 15.06)	0.0204
SF-36 role limitations due to physical problem differences between means (95% CI)	–	19.97 (–0.25, 40.18)	0.0529	21.93 (1.96, 41.89)	0.0314	21.93 (1.96, 41.89)	0.0314	22.42 (2.15, 42.70)	0.0303
SF-36 pain differences between means (95% CI)	–	11.17 (–0.29, 22.63)	0.0561	8.51 (–2.83, 19.84)	0.1407	8.51 (–2.83, 19.84)	0.1407	14.01 (2.52, 25.50)	0.017
SF-36 general health differences between means (95% CI)	–	17.17 (10.08, 24.25)	<0.0001	10.96 (3.96, 17.96)	0.0022	10.96 (3.96, 17.96)	0.0022	15.04 (7.93, 22.14)	<0.0001
SF-36 energy/fatigue differences between means (95% CI)	–	10 (1.93, 18.07)	0.0153	7.24 (–0.76, 15.24)	0.0761	7.24 (–0.76, 15.24)	0.0761	8.58 (0.49, 16.67)	0.0376
SF-36 social function differences between means (95% CI)	–	4.07 (–4.93, 13.08)	0.3744	8.69 (–0.19, 17.57)	0.0552	8.69 (–0.19, 17.57)	0.0552	11.88 (2.85, 20.91)	0.0101
SF-36 emotional well-being differences between means (95% CI)	–	30.13 (13.64, 46.62)	0.0004	12.44 (–3.86, 28.74)	0.1343	12.44 (–3.86, 28.74)	0.1343	21.65 (5.11, 38.19)	0.0104
SF-36 role limitations due to emotional problem differences between means (95% CI)	–	11.08 (3.97, 18.18)	0.0024	7.85 (0.82, 14.88)	0.0288	7.85 (0.82, 14.88)	0.0288	15.04 (7.92, 22.17)	<.0001
SF-36 health change differences between means (95% CI)		17.42 (5.75, 29.08)	0.0035	13.17 (1.66, 24.68)	0.0251	13.17 (1.66, 24.68)	0.0251	20.85 (9.15, 32.55)	0.0005

CI = confidence interval; ITT = intention to treat; SF-36 = Short-Form Questionnaire.

a Pre/post comparison by a mixed-effect model.

Adverse events (AEs) are comprehensively presented in the primary report [17], with further details available at evidence.nejm.org. Of all the participants, a total of 205 AEs in were documented in 101 individuals in the published study [17]. The numbers of AEs reported in the 3-mo and placebo groups relevant to this substudy were 76 (in 34 individuals) and 81 (in 39 individuals), respectively. Only nine out of the 205 AEs were considered as adverse reactions (ARs) to study intervention (ARs were not reported in the primary publication), presented in a total of five participants: two from the placebo and three from the MV140 3-mo group. The nine ARs were expected, delayed, and resolved with no sequelae. Six of these ARs were assessed as local (placebo: one; MV140 3 mo: five) and were mostly mild (itchy and sore mouth or gastric discomfort). Three were classified as grade I–II systemic reactions (placebo: one; MV140 3 mo: two).

## 4 Discussion

The pivotal RCT [17] which this secondary analysis is based on showed a significant reduction of UTIs (from a median six UTIs per year prior to vaccination) in the efficacy period in the individuals treated with 3 mo of MV140 (median 0.0 [IQR 0.0–1.0]) compared with placebo (median 3.0 [IQR 0.5–6.0], *p* < 0.0001). Women with rUTIs treated with 3 mo of MV140 had a UTI-free rate of 56% (95% CI: 44–67%) compared with 25% (95% CI: 15–35%) for the placebo-treated individuals. The primary RCT also reported no safety concerns with the vaccination at both the standard 3-mo dose and the 6-mo dose. The objective of this secondary analysis was to determine the clinical impact of the recommended dose of MV140 on the personal burden of disease in women with rUTIs, using secondary endpoint data from the pivotal RCT evaluating MV140. Based on the observation that there were no clinically relevant differences in efficacy, safety, and even global QoL between the 3- and 6-mo doses in the primary RCT, this subanalysis is focused on the 3-mo dose that will be used in clinical trials going forward and prescribed clinically to patients with rUTIs. The efficacy of the 3-mo dose was presented in the primary publication. Safety was confirmed with these secondary analyses of the placebo and 3-mo dose groups, which included presentation of ARs (not included in the primary publication). While the present analysis is limited by a power calculation based on the UTI outcomes, these secondary analyses of expanded patient burden parameters and QoL domain impact in female patients with rUTIs enrolled in a placebo-controlled RCT add to our understanding of the real impact of reducing the UTI risk. We hypothesized that this safe reduction in UTIs associated with the standard clinical dosing of MV140 would have a favorable impact on both patient burden and QoL compared with placebo.

The symptoms associated with a UTI episode (dysuria, bladder pain, urinary urgency, and frequency) can be extremely uncomfortable and distressing for patients [7], [9], [12]. “The symptom diary questionnaire observations were based on the Likert scale range [21] similar to other symptom scoring questionnaires employed in UTI studies [22].” This analysis shows that MV140 can reduce the overall days with UTI symptoms and total symptoms experienced over time, but it does not reduce the actual symptoms if patients have a breakthrough UTI.

The overall benefit of MV140 must be the overall reduction in UTI episodes and that is reflected in the overall improvement in QoL, as shown in the change in SF-36 total score, pain, general health, and health change subscores.

Women’s daily activities are impacted severely by rUTIs [11]. Each episode of UTI limits personal daily activities, and for many, this results in productivity loss. Time off work or limited activity for 1–2 days per UTI episode [10] with approximately 3 lost workdays per year has been reported [11]. The present study demonstrated that MV140, prescribed at the clinically recommended dose, improved physical function, role limitations due to physical problems, as well as energy/fatigue.

Women’s mental well-being is also impacted severely by rUTIs. The sudden, unforeseeable, and distressing nature of painful UTI episodes often causes some degree of anxiety and/or depression in up to 62% female patients [9]. Mental health scores are reportedly below average in up to 81% of women [11]. Thirty-four (34%) women with rUTIs associate UTIs with a negative impact on their physical sexual intimacy, while 57% believe that the chronic and recurrent nature of the disease negatively influences their other social relationships [8]. Interestingly, treatment with the recommended 3-mo dose of MV140 compared with placebo did not have a significant impact on social function, but improved emotional well-being and role limitations due to emotional problems.

Antibiotics have been the guideline-recommended therapy for rUTIs [3], [4]. While the present study was not designed to document either side effects of antibiotics or their impact on antibiotic resistance, it is well recognized that these effects of antibiotics are also a part of the problem. Apart from the threat of overall antimicrobial resistance in the general population, one of the most problematic issues for patients suffering from rUTIs is the impact of short-term side effects while taking antibiotics and long-term consequences of multiple courses of wide-spectrum antimicrobials [15], [16]. While common side effects can be experienced with such antimicrobial therapy, development of intolerance (or allergies) to the standard antibiotics prescribed for UTI treatment and potential development of a personal reservoir of resistant bacterial pathogens may make treatment of subsequent UTIs even more difficult to manage [13]. The present study indicates that MV140 significantly reduces the number of days on antibiotics in women with rUTIs by approximately 57%. This could be very impactful when considering that patients on placebo received, on average, almost 30 days of antibiotics following European Association of Urology guidelines (which includes option of long-term prophylaxis for rUTIs) [4]. While not confirmed in this study, it is anticipated that MV140 would improve antibiotic stewardship if used for the management of rUTIs.

Ultimately, the benefits of MV140 intervention must be balanced against the risk of this novel sublingual vaccine approach. Individuals administered MV140 for the recommended 3 mo had few ARs to the vaccine; most were mild and self-limited. This vaccine strategy to prevent UTIs and subsequently reduce the personal burden in women suffering from rUTIs appears to be very safe.

## 5 Conclusions

MV140 taken as directed for 3 mo significantly reduces the personal burden of UTI disease by safely decreasing the risk of subsequent UTIs, reducing severity of overall UTI symptoms, reducing antibiotic use, and ultimately improving almost all domains of QoL in women with rUTIs. This nonantibiotic strategy to prevent or reduce UTIs should empower women suffering from rUTIs.

  ***Author contributions*:** J. Curtis Nickel had full access to all the data in the study and takes responsibility for the integrity of the data and the accuracy of the data analysis.

  *Study concept and design*: Nickel, Foley, Yang, Casanovas, Caballero, Diez-Rivero, Lorenzo-Gómez.

*Acquisition of data*: Foley, Yang, Lorenzo-Gómez.

*Analysis and interpretation of data*: Nickel, Foley, Yang, Casanovas, Caballero, Diez-Rivero, Lorenzo-Gómez.

*Drafting of the manuscript*: Nickel, Diez-Rivero.

*Critical revision of the manuscript for important intellectual content*: Nickel, Foley, Yang, Casanovas, Caballero, Diez-Rivero, Lorenzo-Gómez.

*Statistical analysis*: Nickel, Casanovas, Caballero, Diez-Rivero.

*Obtaining funding*: Nickel, Casanovas.

*Administrative, technical, or material support*: Diez-Rivero.

*Supervision*: Nickel.

*Other*: None.

  ***Financial disclosures***: J. Curtis Nickel certifies that all conflicts of interest, including specific financial interests and relationships and affiliations relevant to the subject matter or materials discussed in the manuscript (eg, employment/affiliation, grants or funding, consultancies, honoraria, stock ownership or options, expert testimony, royalties, or patents filed, received, or pending), are the following: J.C. Nickel, S. Foley, B. Yang, and M.-F. Lorenzo-Gómez were unpaid investigators in the primary RCT and publication. J.C. Nickel has received research support from Inmunotek S.L. for an unrelated study. J.C. Nickel and M.-F. Lorenzo-Gómez have been investigators and advisors for Inmunotek. J.C. Nickel has been a consultant for Inmunotek S.L. and OM Pharma. S. Foley and B. Yang have been investigators for Inmunotek. M. Casanovas, R. Caballero, and C.M. Diez-Rivero are employees of Inmunotek S.L.

  ***Funding/Support and role of the sponsor*:** The primary clinical trial and publication were supported by Inmunotek S.L. This secondary analysis is investigator initiated with no external funding. The sponsor played a role in the design and conduct of the study, collection and management of the data, and review of the manuscript.

  ***Acknowledgments*:** We acknowledge the assistance of Francini Ferreira (Bioclever S.L.) for carrying out the statistical analysis.
